# Epidemiology and Survival Outcomes of Lung Cancer: A Population-Based Study

**DOI:** 10.1155/2019/8148156

**Published:** 2019-12-28

**Authors:** Huan-Tang Lin, Fu-Chao Liu, Ching-Yang Wu, Chang-Fu Kuo, Wen-Ching Lan, Huang-Ping Yu

**Affiliations:** ^1^Department of Anesthesiology, Chang Gung Memorial Hospital, Taoyuan, Taiwan; ^2^College of Medicine, Chang Gung University, Taoyuan, Taiwan; ^3^Graduate Institute of Clinical Medical Sciences, College of Medicine, Chang Gung University, Taoyuan, Taiwan; ^4^Division of Thoracic and Cardiovascular Surgery, Department of Surgery, Chang Gung Memorial Hospital, Taoyuan, Taiwan; ^5^Division of Rheumatology, Allergy and Immunology, Chang Gung Memorial Hospital, Taoyuan, Taiwan; ^6^Division of Rheumatology, Orthopaedics and Dermatology, University of Nottingham, Nottingham, UK; ^7^Center for Big Data Analytics and Statistics, Chang Gung Memorial Hospital, Taoyuan, Taiwan; ^8^Department of Anesthesiology, Xiamen Chang Gung Hospital, Xiamen, China

## Abstract

**Purpose:**

Lung cancer has been the top-ranking cause of cancer deaths in Taiwan for decades. Limited data were available in global cancer surveillance regarding lung cancer epidemiology in Taiwan, and previous reports are outdated.

**Patients and Methods:**

This population-based cohort study extracted data of patients with lung cancer from the Taiwan National Health Insurance database and determined the lung cancer incidence and prevalence during 2002–2014. Histological subtypes were retrieved from the Taiwan Cancer Registry database; survival rates were gathered from the National Death Registry. Average annual percentage changes (APCs) of prevalence, incidence, and overall survival were estimated by joinpoint regression analysis.

**Results:**

Age-standardized incidence of lung cancer increased from 45.04 per 100,000 person-years in 2002 to 49.86 per 100,000 person-years in 2014, with an average APC of 0.7 (95% CI = 0.3–1.1; 0.2 in males, 2.0 in females). Lung cancer was more prevalent in male patients, but this increase gradually slowed down. Socioeconomic analysis showed that lung cancer has higher prevalence in patients with higher income level and urban residency. Adenocarcinoma was the most abundant histological subtype in Taiwan (adenocarcinoma-to-squamous cell carcinoma ratio = 4.16 in 2014), with a 2.4-fold increase of incident cases during 2002–2014 (from 43.47% to 64.89% of all lung cancer cases). The 5-year survival rate of lung cancer patients in 2010 was 17.34% (12.60% in male, 25.40% in female), with an average APC of 9.3 (6.3 in male, 11.8 in female) during 2002–2010.

**Conclusion:**

Average APCs of prevalence and incidence of lung cancer were 3.1 and 0.7, respectively, during 2002–2014 in Taiwan. The number of female patients and number of patients with adenocarcinoma have increased the most, with incident cases doubling in these years. Facing this fatal malignancy, it is imperative to improve risk stratification, encourage early surveillance, and develop effective therapeutics for lung cancer patients in Taiwan.

## 1. Introduction

Lung cancer is the top-ranking cause of cancer deaths worldwide, and the incidence has risen over the last three decades. According to the latest GLOBOCAN 2018 estimates, lung cancer is the most often diagnosed malignancy (2.1 million new cases, equal to 11.6% of the total incident cancer cases in 2018) with an age-standardized incidence rate of 22.5 (31.5 in male, 14.6 in female) per 100,000 person-years worldwide in 2018 [1]. Furthermore, lung cancer ranked first and accounted for 18.4% of the total cancer deaths (equal to 1.8 million deaths) in 2018, with an age-standardized mortality rate of 18.6 (27.1 in males, 11.2 in females) per 100,000 persons [2]. In 2015, lung cancer accounted for 36.4 million (95% uncertainty interval, 35.4–37.6 million) disability-adjusted life-years and ranked first among all malignancies for absolute years of life lost in both sexes [3]. Considering absolute cases, lung cancer accounts for most of the cancer incident cases and deaths in the majority of developed countries; however, the incidence and mortality are lower (ranking tenth and seventh, respectively) in countries with a lower sociodemographic index [3]. In the CONCORD-3 program, a global surveillance of cancer survival in 71 countries, the 5-year survival for patients diagnosed with lung cancer during 2010–2014 was high in Japan (32.9%), followed by 20–30% in another 12 countries such as Korea (25.1%) and the USA (21.2%); the range was 10–19% in other countries [4].

Generally, men have a higher chance of developing lung cancer than women. However, the incidence rates of male patients have fallen since the mid-1990s in most developed countries, while the incidence rates of female patients have continuously increased [5]. Therefore, the incidence rates of lung cancer in male and female patients have converged in the USA and several other developed countries, especially among the younger generation [5, 6]. The differential incidence based on sex might be contributed by sex-specific differences in histological subtypes of lung cancer as well as the change of prevalence in smoking.

Typical risk factors for lung cancer include tobacco smoking, family history of malignancy, previous lung diseases, and exposure to secondhand smoke, radon, asbestos, arsenic, air pollutants, or occupational carcinogens [5, 7]. The different subtypes of lung cancer possess distinct epidemiological and prognostic features [8]. Smoking has been identified as the major risk factor for small cell lung cancer (SCLC) and squamous cell carcinoma. More than 80% of lung cancer etiology could be ascribed to smoking in the Western population. The occurrence of lung cancer has been reduced through tobacco control such as increasing tobacco taxes and prices, health warnings on packages, and advocating smoking cessation, as well as through comprehensive ban on tobacco advertising [1]. On the other hand, adenocarcinoma is more common in the Asian population, particularly among females and never-smokers [5, 9]. Robust data exist regarding the prevalence of *EGFR* mutations in adenocarcinoma patients, ranging from the highest *EGFR* mutation frequency of 47% in the Asia-Pacific subgroup to the lowest frequency of 12% in the Oceania subgroup [10]. Studies in China and Hong Kong suggested that the rise of lung adenocarcinoma in Asian nonsmokers has significant exposure-response relationships with secondhand smoke and cooking fumes [11, 12].

Population-based cancer registries are essential for assessing the current cancer burden of the healthcare systems, as well as to monitor the efficacy of cancer prevention and treatment strategies. Regarding lung cancer epidemiology in Taiwan, limited data were available in global cancer surveillance and information reported in previous studies has become outdated [13, 14]. In this population-based epidemiologic study, we managed to update the trends of the prevalence, incidence, and overall survival of patients with lung cancer in Taiwan between 2002 and 2014 by combining information from the National Health Insurance Research Database (NHIRD), Taiwan Cancer Registry Database (TCRD), and National Death Registry (NDR).

## 2. Materials and Methods

### 2.1. Source Data and Study Population

The Taiwan NHIRD has been regularly collecting demographic data on the diagnoses, prescriptions, and operations of all Taiwan National Health Insurance (NHI) beneficiaries from primary care and special care providers since 1995. The NHI is a universal health insurance that had an extensive coverage of over 99.6% of registered beneficiaries in Taiwan at the end of 2014 [15]. Individual information contained in the NHI database during 2002–2014 was encrypted utilizing the International Classification of Diseases, Ninth Revision, Clinical Modification (ICD-9-CM) code. The NHIRD is a large representative health care database, and its validity and clinical consistency in cancer research have been proved [16]. This population-based epidemiologic study was reviewed and permitted by the Institutional Review Board of Chang Gung Medical Foundation, Taiwan (approval number: 201801054B1). As the source data used in this study were totally deidentified and encrypted, the requirement for obtaining patient consent was waived.

This study utilized the crosslinking databases of the NHIRD, TCRD, and NDR with the approval of the Department of Statistics, Ministry of Health and Welfare of Taiwan. The utilized databases of NHIRD, TCRD, and NDR all belong to subsets of the NHI database. Using a unique personal encrypted identification number for each beneficiary in Taiwan, we are able to crosslink efficiently among different subsets of the NHI database. The internal linkage between each dataset is robust, and the data quality was accepted with validity in previous publications [16, 17]. This population-based cohort study comprised ethnic Taiwanese patients with the main diagnosis of lung cancer (ICD-9-CM code 162.xx) identified via the NHIRD from 2002 to 2014. The histological subtypes of lung cancer were retrieved from the TCRD, while the survival rates were extracted from the NDR database.

### 2.2. Estimate of the Prevalence, Incidence, and Overall Survival

The crude prevalence rate of lung cancer per 100,000 persons was calculated by dividing the number of prevalent lung cancer patients by the eligible population in a specified year. We defined lung cancer patients as individuals who were diagnosed as lung cancer, ICD-9-CM code 162.xx, before July 1 of that calendar year. The eligible population comprised every individual who registered on July 1 of that calendar year. On the other hand, the crude incidence rate of lung cancer per 100,000 person-years was estimated by dividing the number of incident lung cancer patients by the total person-years in the at-risk population during a specified year. The incident lung cancer cases were defined as patients with a record of lung cancer in a specified year but without diagnosis of lung cancer prior to January 1 of that year. People with no history of lung cancer during the same year were defined as the at-risk population. All eligible subjects were followed up from January 1 of the year when the earliest diagnosis of lung cancer was done to the primary outcome of death or the end of the study period on December 31, 2014. To diminish the fundamental restriction of database estimation, the eligible cohorts included patients with registration period more than one-year prior to January 1 of each year. The age-standardized prevalence and incidence rate of lung cancer in each year from 2002 to 2014 were calculated with reference to the population structure of 2014. The prevalence and incidence of lung cancer were further analyzed by dividing patients into subgroups based on sex, calendar year, socioeconomic status, and histological subtypes. The histological subtype of lung cancer was categorized according to the classification of lung cancer in 2004 World Health Organization (WHO) criteria with mainly SCLC and non-small cell lung cancer (NSCLC) including adenocarcinoma, squamous cell carcinoma, large cell carcinoma, and other subtypes [8]. Overall survival rates of patients with lung cancer were extracted from overall mortality rates in the National Death Registry of Taiwan and were relative survival rates by definition. The relative survival rate represents the cumulative probability of a cancer patient who would have survived a given period compared to the comparable general population after adjusting for age, sex, and observed calendar year [18]. The presented 1-year, 3-year, and 5-year survival rates in each calendar year were estimated using the life table method [19]. The geographic variations in the prevalence and incidence of lung cancer in 2002 and in 2014 were also compared by dividing Taiwan into 21 administrative districts, including Keelung, Taipei city, New Taipei City, Taoyuan city, Hsinchu, Miaoli, Taichung city, Changhua, Yunlin, Nantou, Chiayi, Tainan city, Kaohsiung city, Pingtung, Yilan, Hualien, Taitung, Lianjiang, and offshore Penghu islets. The age-standardized prevalence and incidence of lung cancer for each district were estimated with reference to the overall population structure of 2014 to diminish the regional effects of diverse age and sex. To evaluate the recognized risk factors of lung cancer, we also obtained air pollution information including Pollutant Standards Index (PSI) and fine particulate matter (PM 2.5) concentrations from the Taiwan Air Quality Monitoring Network and retrieved publications on the prevalence of smoking from the Adult Smoking Behavior Surveillance System [20–22].

### 2.3. Statistical Analysis

The 95% confidence intervals (95% CIs) for the prevalence, incidence, and survival rate of lung cancer were estimated using Poisson regression. The secular trends for the prevalence, incidence, and survival rates of lung cancer were estimated using the joinpoint regression analysis program (version 4.4.0.0) to generate different “joinpoints” for the secular change and calculate the average annual percentage change (APC) for each linear segment. The Charlson Comorbidity Index (CCI) score was utilized to estimate the medical burden and mortality risk of lung cancer patients. Statistically, continuous variables such as age and CCI score were compared with the *t*-test, while categorical variables such as sex and income level were compared with *χ*^2^ analysis. The statistical significance level in this study was set at *α* < 0.05. All statistical analyses in this study were calculated using the SAS software (version 9.4; SAS Institute, Cary, NC, the United States).

## 3. Results

### 3.1. Demographic Characteristics and Geographic Variations

The total eligible population in our study consisted of 23,850,842 registered NHI beneficiaries (50.02% male, 49.98% female) in 2014. Among these, we identified 124,148 patients with a diagnosis of lung cancer (79,145 males and 45,003 females) during 2002–2014. The demographic characteristics of these lung cancer patients are listed in Table 1. Although the majority of lung cancer patients were male, the percentage of female patients increased from 33.24% in 2002 to 41.07% in 2014. On average, male patients with lung cancer were older (69.58 ± 12.28 years in male versus 66.67 ± 13.35 years in female) and had a lower socioeconomic status (lower income level, urban residency, and professional occupation). The incident cases of lung cancer increased from 7,308 in 2002 to 11,784 in 2014, and the average CCI score at lung cancer diagnosis decreased from 4.23 ± 3.12 to 3.48 ± 3.03. Most lung cancer patients in Taiwan lived in the high-income urban areas during this period. The socioeconomic analyses implied that there was a growing trend towards a higher socioeconomic status among the lung cancer patients in 2014 in comparison with the distribution in 2002.

**Figure 1:**
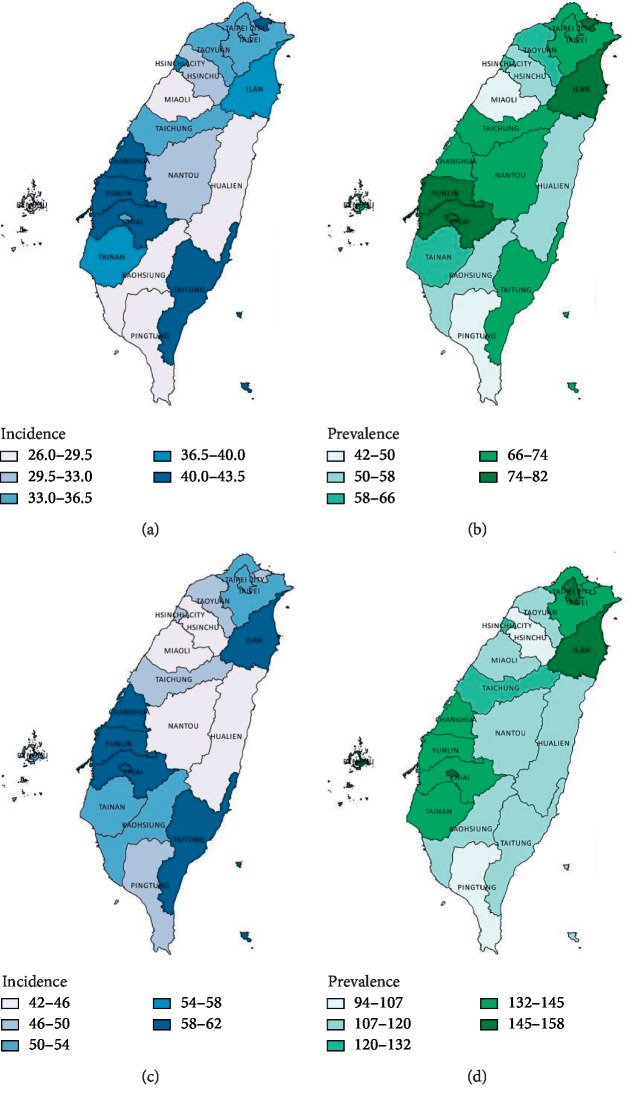
Geographic variation in the prevalence and incidence of lung cancer in Taiwan in 2002 and 2014. (a) Prevalence in 2002. (b) Incidence in 2002. (c) Prevalence in 2014. (d) Incidence in 2014.

**Figure 2:**
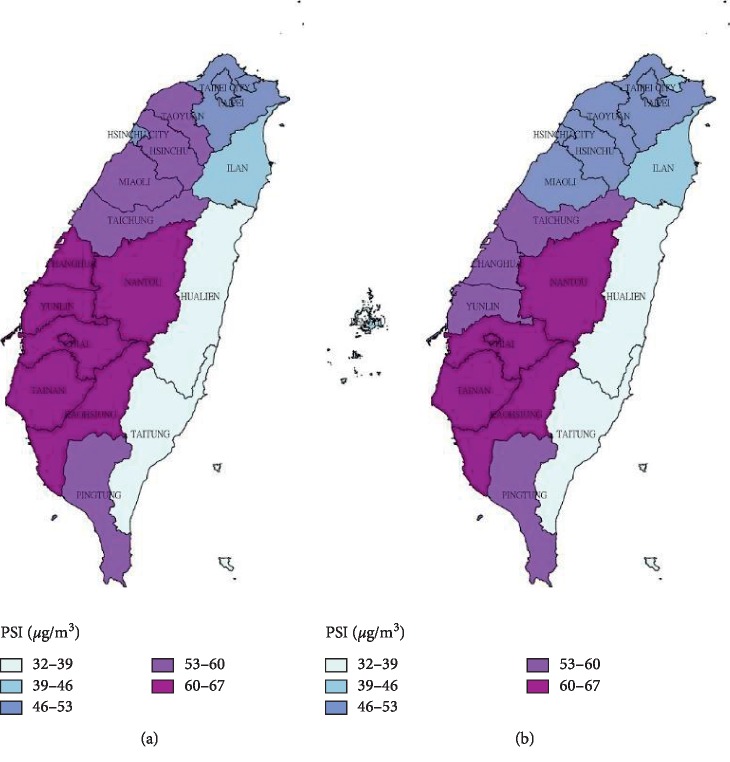
Annual average of Pollutant Standards Index (PSI, *μ*g/m^3^) in Taiwan by county in 2002 and 2014. Data were obtained from the Taiwan Air Quality Monitoring Network. PSI is calculated by averaging air pollutant concentration collected for the past 24 hours including particulate matter (PM10), sulfur dioxide (SO_2_), nitrogen dioxide (NO_2_), carbon monoxide (CO) and ozone (O_3_). PSI >100 is considered to impair health.

**Figure 3:**
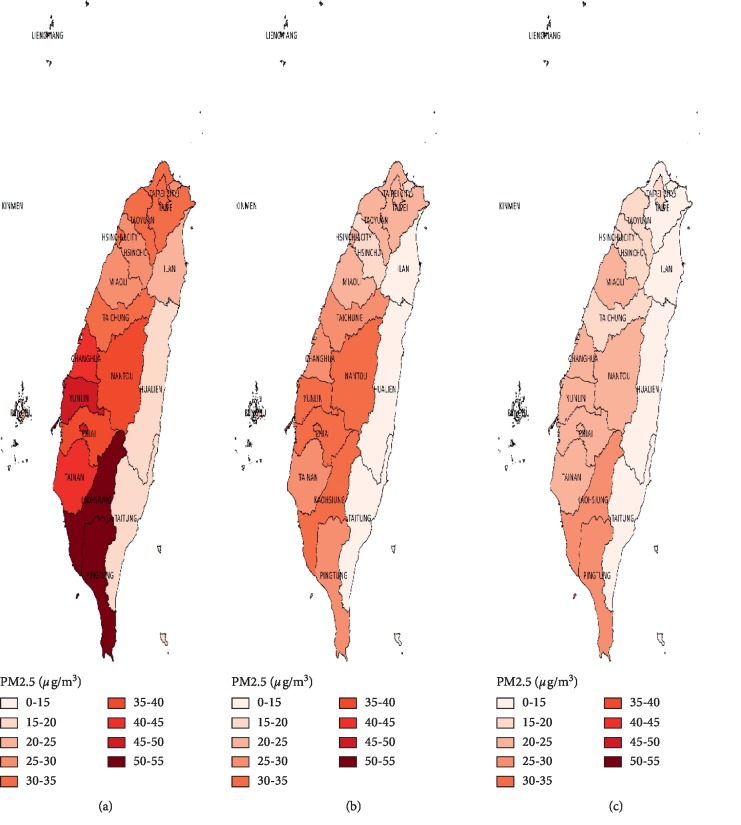
Annual average of fine particulate matter exposure (PM2.5, *μ*g/m^3^) in Taiwan by county in 2005, 2014, and 2018. Data were obtained from the Taiwan Air Quality Monitoring Network. The first nationwide PM2.5 concentration in Taiwan was released since 2005. For counties with several monitoring stations, we selected the monitoring station with the representative county level of air pollution.

The geographic distribution of the prevalence and incidence of lung cancer in 2002 and 2014 is shown in Figure 1. We also demonstrated the change of available air pollution indicators (PSI in 2002 and 2014; PM2.5 in 2005, 2014, and 2018) in every district of Taiwan for possible exposure-disease relationship (PSI in Figure 2; PM2.5 in Figure 3) [20, 21]. Eastern areas of Taiwan showed lower air pollution level in comparison with higher PM2.5 concentrations in southwestern areas. However, this distribution of air pollution indicators could not fully explain the geographic variation of the prevalence and incidence of lung cancer.

### 3.2. Prevalence and Incidence of Lung Cancer

**Figure 4:**
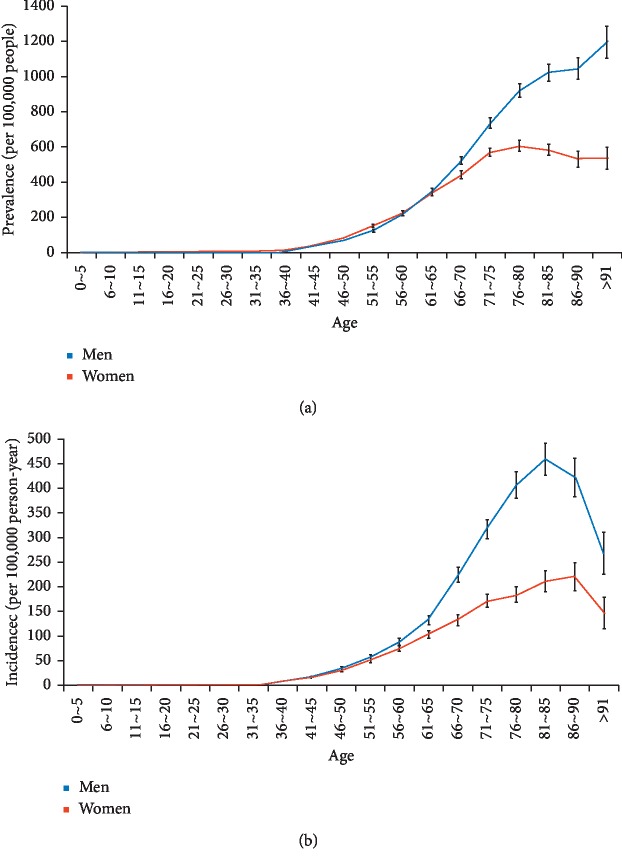
Age-specific (a) prevalence and (b) incidence of lung cancer in Taiwan in 2014 (blue: men; red: women).

**Figure 5:**
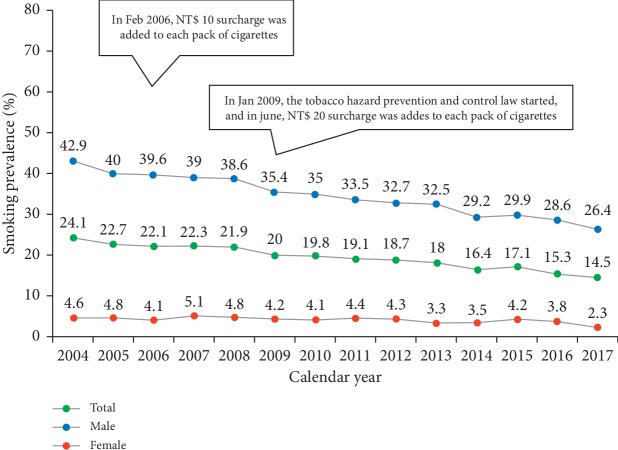
Sex-specific prevalence of smoking among adults (over 18 years of age) in Taiwan during 2004–2017 (green: total population; blue: male; red: female). (1) Data based on adult smoking behavior survey provided by the Taiwan health promotion administration. (2) Current smokers were defined as those who had smoked more than 100 cigarettes (5 packs) and had smoked within the past 30 days. (3) The adult smoking rate was weighted and standardized according to sex, age, educational level, and geographic region in year 2000.

Tables 2 and 3 show the prevalence and incidence of lung cancer in Taiwan during 2002–2014. Generally, a higher prevalence and incidence was observed among the male than female patients with lung cancer during 2003–2014; however, the prevalence and incidence in female patients increased more quickly while the increase in male patients slowed down. The age-standardized prevalence of lung cancer was 93.38 (95% CI = 91.84–94.92) per 100,000 persons in 2002 and 132.40 (95% CI = 130.94–133.86) per 100,000 persons in 2014. The joinpoint analysis of lung cancer prevalence showed that the average APC was 3.1 (95% CI = 2.7–3.4) during 2002–2014, with faster accumulation in the female (average APC, 5.0 (95% CI = 4.8–5.1)) than male patients (average APC, 1.7 (95% CI = 1.3–2.0)) (Table 4). The standardized incidence of lung cancer was 45.04 (95% CI = 43.95–46.13) per 100,000 person-years in 2002 and 49.86 (95% CI = 48.96–50.76) per 100,000 person-years in 2014, with increasing female-to-male incidence ratio from 0.56 in 2002 to 0.69 in 2014. The joinpoint analysis of lung cancer incidence showed the average APC during 2002–2014 was 0.7 (Table 5). Notably, the trend of incidence changes in the male patients demonstrated a diverse direction with upward segment during 2002–2006 (average APC, 2.2 (0.5–4.0)) and downward segment during 2006–2014 (average APC, −0.8 (−1.3 to −0.3)). Overall, the age-standardized prevalence of lung cancer was 1.42-fold higher in 2014 than in 2002; similarly, the age-standardized incidence of lung cancer was 1.11-fold higher in 2014 than in 2002. The age-specific prevalence and incidence of lung cancer in 2014 is shown in Figure 4. The majority of lung cancer patients (95.22%) were diagnosed at an age ≥45 years. Interestingly, the age-specific prevalence of lung cancer was slightly higher in the female than male patients aged ≤60 years, whereas the age-specific incidence of lung cancer was predominant in male patients, except those aged 25–35 years.  Figure 5 shows the sex-specific differences in the prevalence of smoking among adults in Taiwan during 2004–2017 [22]. The overall prevalence of smoking among adults decreased gradually from 24.1% in 2004 to 14.5% in 2017 as a result of a concurrent 38.5% decrease in prevalence of smoking among men; the prevalence of smoking among women, however, persisted at around 4% during these years.

### 3.3. Analysis of Lung Cancer Overall Survival Rates

**Figure 6:**
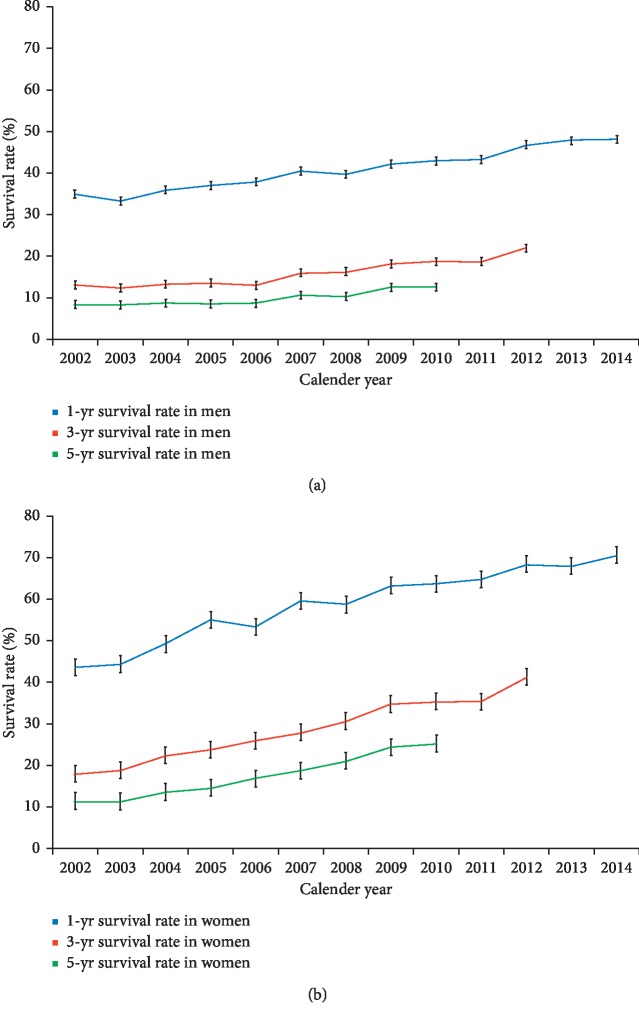
Secular trends of 1-year, 3-year, and 5-year survival rates of lung cancer in (a) men and (b) women in Taiwan from 2002 to 2014 (blue: 1-year survival rate; red: 3-year survival rate; green: 5-year survival rate).

Joinpoint analysis of overall lung cancer survival trends by sex and CCI in Taiwan are shown in Table 6. The 1-, 3-, and 5-year survival rates of lung cancer in Taiwan improved significantly in either sex, especially in the female patients with an average APC of 11.8 (95% CI = 10.5–13.0) for the 5-year survival rate during 2002–2010 (also see Figure 6). Despite these improvements, the 5-year survival rate in 2010 was merely 17.34% (12.60% in males, 25.40% in females). The male lung cancer patients had a relatively poor prognosis with 1-, 3-, and 5-year survival rates of 48.20% in 2014, 22.00% in 2012, and 12.60% in 2010, respectively, compared with 1-, 3-, and 5-year survival rates of 70.60%, 41.10%, and 25.40%, respectively, in the females. To evaluate the influence of comorbidities on the overall survival rates, we stratified patients into two groups according to the calculated CCI score at lung cancer diagnosis. Lung cancer patients with a CCI score ≤1 (28.7% of total lung cancer patients) had much better prognosis with a 5-year survival rate of 24.5% in 2010 compared with a mere 14.2% in patients with a CCI score ≥2 (71.3% of total lung cancer patients).

### 3.4. Analysis Based on the Histological Subtypes of Lung Cancer

Table 7 shows the demographic characteristics of patients with different histological subtypes of lung cancer. Patients with lung adenocarcinoma comprised 54.49% of total lung cancer patients during 2002–2014 and the highest proportion of female patients (49.64%); moreover, these patients had a higher socioeconomic status (urban residency, higher income level, and professional occupation) compared with patients with other subtypes of lung cancer. On the other hand, patients with squamous cell carcinoma and SCLC were presented at an older age (70.89 ± 11.13 and 69.90 ± 11.00 years); had more comorbidities at diagnosis (CCI: 3.58 ± 2.86 and 4.02 ± 3.14); were less likely to be a female (12.81% and 10.87%); and were less likely to be categorized into high socioeconomic status among these subtypes.

**Figure 7:**
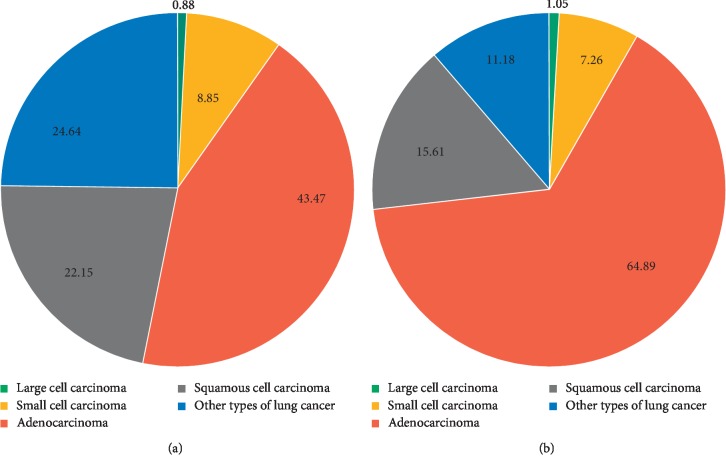
Percentage of different histological subtypes of lung cancer in Taiwan in (a) 2002 and (b) 2014 (red: adenocarcinoma; gray: squamous cell carcinoma; blue: other types of lung cancer; yellow: small cell carcinoma; green: large cell carcinoma).

**Figure 8:**
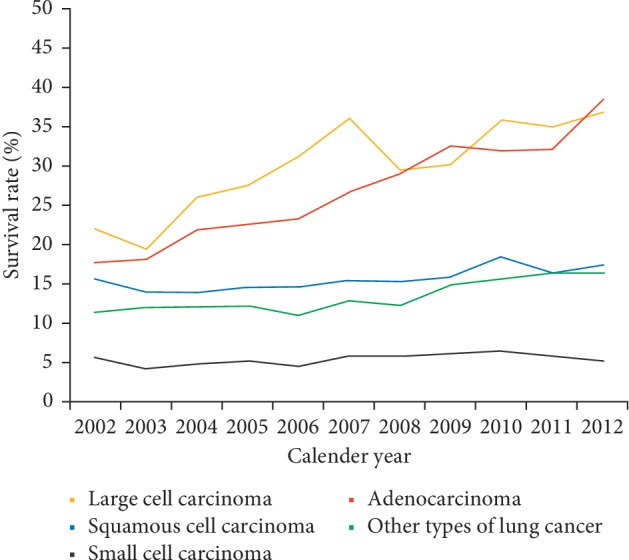
Secular trends of 3-year survival rate in patients with different subtypes of lung cancer (orange: large cell carcinoma; red: adenocarcinoma; blue: squamous cell carcinoma; green: other types of lung cancer; gray: small cell carcinoma).

Figure 7 illustrates the incident percentage of different subtypes of lung cancer in Taiwan in 2002 and 2014. Adenocarcinoma was the most abundant subtype of lung cancer, growing from 43.47% of the total cases in 2002 (3,177 incident cases) to 64.89% in 2014 (7,647 incident cases). Meanwhile, the percentage of squamous cell carcinoma and SCLC decreased substantially during 2002–2014 (squamous cell carcinoma, 22.15% in 2002 to 15.61% in 2014; SCLC, 8.85% in 2002 to 7.26% in 2014).  Table 8 shows the variation in overall survival rates in different subtypes of lung cancer. In a joinpoint analysis, the 5-year survival rate in 2010 for large cell carcinoma, adenocarcinoma, squamous cell carcinoma, other subtypes, and small cell carcinoma was 30.2%, 22.0%, 12.9%, 11.6%, and 4.4%, respectively. Among these subtypes, the survival rates of adenocarcinoma improved most with an average APC of 10.6 (95% CI = 8.4–12.9) for a 5-year survival rate during 2002–2010. The secular trends of 3-year overall survival for patients with different subtypes (Figure 8) revealed that large cell carcinoma had a relative better survival rate, adenocarcinoma improved the most, and SCLC had a relatively poor prognosis.

## 4. Discussion

In this population-based cohort study, which is mainly based on the NHIRD, the temporal change of lung cancer epidemiology in Taiwan during 2002–2014 was evaluated thoroughly by correlating with the histological subtypes and overall survival rates. The age-standardized incidence rate of lung cancer in Taiwan was 49.86 per 100,000 person-years in 2014, which is lower than the reported incidence rate in Europe and North America, but the trend of incidence kept rising with an average APC of 0.7 (95% CI 0.3–1.1) during 2002–2014 [5]. A higher incidence of lung cancer was observed in the males; however, the incidence in the females increased more rapidly than that in the males, with the female-to-male incidence ratio increasing from 0.56 in 2002 to 0.69 in 2014. Besides, we also observed that lung cancer developed more frequently in patients with a higher income level and urban residency. The urban-rural disparity might be correlated with a higher air pollution and environmental smoke exposure in the urban areas. Adenocarcinoma comprised almost two-thirds of lung cancer patients in 2014, and the survival rates of patients with adenocarcinoma improved the most during 2002–2014. Because of the accelerated incidence and improved overall survival, adenocarcinoma is becoming the increasingly predominant histological subtype of lung cancer in Taiwan. Following the one-third decrease in the prevalence of smoking among adults in this period, the percentage of smoking-correlated subtypes (squamous cell carcinoma and SCLC) also declined substantially. The 5-year survival rate of lung cancer was 17.34% in 2010, but it improved a lot with an average APC of 9.30 during 2002–2010, especially in the female patients and patients with the adenocarcinoma subtype.

SCLC is strongly associated with cigarette smoking and, consequently, it is deemed as highly preventable. The decreased incidence of SCLC in the United States over the past 30 years could be explained by the decrease in the prevalence of smoking, particularly among men, and by the decrease in the percentage of tar and nicotine in cigarettes [23]. Based on the increasing health and economic burden of tobacco use, the Taiwan Ministry of Health and Welfare implemented the Tobacco Hazards Prevention Act in 1997 and has put forward a sequential Health and Welfare Surcharge since 2002 [24]. The Taiwan government also joined the global fight against tobacco by participating in the Framework Convention for Tobacco Control under the auspices of the WHO in 2005 and endeavored to implement the MPOWER package [24]. To monitor the effects of the tobacco control legislations, the Taiwan Health Promotion Administration has been conducting an annual national telephone survey on smoking behavior since 2004 [22]. Ever since, a significant decrease in the prevalence of smoking has been observed in Taiwan, mainly among the middle-aged men; we have also observed a significant decrease in smoking-correlated subtypes of lung cancer in our study. Although initial success has been achieved in reducing the prevalence of cigarette smoking, efforts to promote smoking prevention, encourage smoking cessation, and reduce secondhand smoke exposure remain our top priority [25].

Most women with lung cancer are nonsmokers, however, and the incidence of lung adenocarcinoma became increasingly predominant, especially in the Asian population [9]. Indoor and outdoor air pollution has been deemed as a significant contributor for lung cancer, especially for lung adenocarcinoma and female patients; additionally, it might play a role in the observed urban-rural disparity of lung cancer in Taiwan [26]. The exposure-response relationship was demonstrated by several large cohorts where each 10 *μ*g/m^3^ increase in the ambient PM 2.5 concentration was correlated with an increased risk of lung adenocarcinoma (hazard ratio 1.31; 95% CI = 0.87–1.97) in nonsmokers and an additional 15–27% risk of lung cancer mortality in lifelong never-smokers [26–28]. Former smokers had an additional risk of lung cancer in association with outdoor particular matter exposure compared with never-smokers [29]. Therefore, the International Agency for Research on Cancer announced in 2013 that outdoor air pollution exposure, especially particulate matter, as a Group 1 carcinogen to human, especially for lung cancer [26]. The deteriorating air pollution in the Asian countries after decades of rapid industrialization and urbanization has been recognized as the worst region among the world [30]. The potential health impacts of air pollution are multisystemic, and several studies had established the causal link of PM2.5 with lung cancer, ischemic heart disease, stroke, and pulmonary diseases [31]. Taiwan has established a nationwide air quality monitoring system and has been releasing a monthly report of PM2.5 concentration since 2005 [21]. The PM2.5 distribution was generally higher in central and southwestern Taiwan possibly due to widespread coal-fired power plants and heavy industries. Although the PM2.5 concentration in most areas of Taiwan has shown decreasing trends in recent years along with other air pollutants, the estimated national average of annual PM2.5 concentration in 2017 (20.7 *μ*g/m^3^) was still far above the optimal level recommended by WHO air quality guideline (10 *μ*g/m^3^) [21, 32]. Since the consequence of air pollution is far-reaching and long-lasting, our government should set more stringent air pollution regulations and enforce environmental protection for benefit of the next generation.

Delayed diagnosis is the major reason behind poor long-term survival of lung cancer patients, as most patients were at an advanced stage at first diagnosis which made early interventions almost impossible. Regarding surveillance of lung cancer, low-dose computed tomography (CT) was effective in identifying lung cancer at early stage. The National Lung Screening Trial conducted in the US deduced that screening with the use of low-dose CT reduced 20.0% of lung cancer-specific mortality compared with chest radiography in high-risk smokers (aged 55–75 years with smoking intensity ≥30 pack-years) [33]. Therefore, low-dose CT has already been included under insurance coverage for screening of lung cancer by the Centers for Medicare and Medicaid Services in the US since 2015 [34]. In Taiwan, the majority of lung cancer cases identified by the use of low-dose CT screening were adenocarcinoma at early stage. Thus, implementation of CT screening in Taiwan would be more cost-effective than in the Western countries where adenocarcinoma is less prevalent [35, 36]. Radiation exposure and risk of malignancy arising from the use of low-dose CT screening for lung cancer might be a major concern, but many screening trials showed that these related risks can be considered acceptable and even negligible considering the associated substantial mortality reduction [37]. With accessible health checkup services and an appropriate screening program for the high-risk population, the use of low-dose CT screening might identify more patients with early stage, potentially curable lung cancer for timely treatment and thereby improve the overall prognosis [38].

Strengths of this study include an extended 13-year follow-up period (2002–2014) with a total of 124,148 observed lung cancer patients; representative data extracted from the nationwide database of the NHIRD, the TCRD, and NDR; detailed analyses of socioeconomic status, comorbidities, histological subtypes, and geographic variations; and the availability of ecologic measures of plausible risk factors including air pollution concentration and smoking prevalence. Therefore, our updated reports on lung cancer epidemiology are comprehensive and trustworthy for the Taiwanese population. The epidemiological results presented here provide valuable information for the development of future healthcare policies in order to reduce the huge burden of lung cancer.

There were several limitations and uncertainties in our study. First, clinical information on stages, treatment, and molecular profiling of lung cancer were not presented in this study due to the limitations of the available databases. Therefore, it became inaccessible to evaluate the possible impact of early surveillance, novel therapies, and other prognostic factors on overall survival of lung cancer. Nevertheless, we have managed to include the latest available evidences to present a comprehensive lung cancer epidemiology in Taiwan. Second, the associations between known carcinogens and the incidence or mortality rate of lung cancer are difficult to evaluate based on available information. For example, our presented air pollution indicators (PSI, PM 2.5) might provide partially explanation for the higher prevalence and incidence of lung cancer in the southwestern Taiwan. But the other geographic variation of lung cancer distribution requires further investigation on other causative factors such as smoking, environmental carcinogens, or subpopulation susceptibility. Third, coding errors might occur in large national databases despite routine checkup, thus leading to possible underestimation of the incidence and survival rates of lung cancer.

## 5. Conclusion

This nationwide cohort study of lung cancer in Taiwan found that the incidence and prevalence of lung cancer increased 1.11-fold and 1.42-fold, respectively, with almost two-thirds of patients with lung cancer belonging to subtype of adenocarcinoma in 2014. The slowing down of the incidence of lung cancer in male patients and decrease of smoking-correlated subtypes (SCLC and squamous cell carcinoma) might be attributed to the dramatic decrease in the prevalence of smoking in adults, especially among the middle-aged males. The overall survival rates of lung cancer were relatively poor, but it improved a lot in these years, especially in female patients and patients with adenocarcinoma. The Taiwan government has implemented sequential strategies to alleviate the health and economic burden of lung cancer, including tobacco control regulations, reduction of air pollution, application of sophisticated therapeutics, and early surveillance with low-dose CT screening. Despite these efforts, the incidence of lung cancer in Taiwan was still on the rise and the attributable cancer deaths were the highest among all malignancies. Therefore, we should dedicate more resources to improve the prevention strategies, encourage early surveillance, and develop effective therapeutics for lung cancer patients in Taiwan.

## Figures and Tables

**Table 1:** Clinical characteristics of lung cancer patients in Taiwan from 2002 to 2014.

	Entire cohort (*n* = 124,148)		By gender					By calendar year				
			Female (*n* = 45,003)		Male (*n* = 79,145)		*p* value	2002 (*n* = 7,308)		2014 (*n* = 11,784)		*p* value
Age (years) (mean ± standard deviation)	68.53 ± 12.75		66.67 ± 13.35		69.58 ± 12.28		<0.0001^*∗*^	68.13 ± 12.30		67.80 ± 12.90		0.0762
Sex							—					<0.0001^*∗*^
Female	45,003	(36.25)	—	—	—	—		2,429	(33.24)	4,840	(41.07)	
Male	79,145	(63.75)	—	—	—	—		4,879	(66.76)	6,944	(58.93)	
Place of residence, no. (%)							<0.0001^*∗*^					<0.0001^*∗*^
Urban	62,187	(50.09)	24,468	(54.37)	37,719	(47.66)		3,609	(49.38)	6,597	(55.98)	
Suburban	36,873	(29.70)	12,670	(28.15)	24,203	(30.58)		2,353	(32.20)	3,744	(31.77)	
Rural	14,900	(12.00)	4,951	(11.00)	9,949	(12.57)		1,037	(14.19)	1,385	(11.75)	
Unknown	10,188	(8.21)	2,914	(6.48)	7,274	(9.19)		309	(4.23)	58	(0.49)	
Income levels, no. (%)							<0.0001^*∗*^					<0.0001^*∗*^
Quintile 1	16,536	(13.32)	4,747	(10.55)	11,789	(14.90)		1,924	(26.33)	0	(0.00)	
Quintile 2	31,632	(25.48)	11,466	(25.48)	20,166	(25.48)		3,241	(44.35)	3,141	(26.65)	
Quintile 3	16,190	(13.04)	5,758	(12.79)	10,432	(13.18)		184	(2.52)	597	(5.07)	
Quintile 4	26,534	(21.37)	10,586	(23.52)	15,948	(20.15)		485	(6.64)	5,075	(43.07)	
Quintile 5	23,469	(18.90)	9,682	(21.51)	13,787	(17.42)		1,096	(15.00)	2,931	(24.87)	
Unknown	9,787	(7.88)	2,764	(6.14)	7,023	(8.87)		378	(5.17)	40	(0.34)	
Occupation, no. (%)							<0.0001^*∗*^					<0.0001^*∗*^
Dependents of the insured individuals	36,796	(29.64)	16,724	(37.16)	20,072	(25.36)		2,050	(28.05)	4,172	(35.40)	
Civil servants, teachers, military personnel, and veterans	5,876	(4.73)	1,617	(3.59)	4,259	(5.38)		160	(2.19)	1,132	(9.61)	
Nonmanual workers and professionals	8,990	(7.24)	3,491	(7.76)	5,499	(6.95)		474	(6.49)	1,213	(10.29)	
Manual workers	38,813	(31.26)	14,032	(31.18)	24,781	(31.31)		28,68	(39.24)	3,926	(33.32)	
Other	33,673	(27.12)	9,139	(20.31)	24,534	(31.00)		1,756	(24.03)	1,341	(11.38)	
Charlson Index (mean ± standard deviation)	3.80 ± 3.09		3.73 ± 3.11		3.83 ± 3.08		<.0001^*∗*^	4.23 ± 3.12		3.48 ± 3.03		<0.0001^*∗*^

^*∗*^ *p*  < 0.05.

**Table 2:** Crude and age-standardized prevalence (per 100,000 persons) of lung cancer in Taiwan from 2002 to 2014.

Year	Total					Male					Female				
	*N*	Crude		Standardized		*N*	Crude		Standardized		*N*	Crude		Standardized	
2002	22,458,027	69.12	(68.03–70.21)	93.38	(91.84–94.92)	11,381,756	87.61	(85.89–89.32)	114.95	(112.56–117.35)	11,076,271	50.13	(48.81–51.44)	71.79	(69.85–73.74)
2003	22,666,986	70.57	(69.48–71.67)	93.44	(91.93–94.95)	11,488,565	88.90	(87.17–90.62)	114.74	(112.40–117.09)	11,178,421	51.74	(50.41–53.08)	72.12	(70.21–74.03)
2004	22,823,802	75.84	(74.71–76.97)	98.19	(96.67–99.71)	11,560,132	94.69	(92.91–96.46)	119.73	(117.39–122.08)	11,263,670	56.49	(55.10–57.88)	76.62	(74.69–78.55)
2005	22,955,392	81.10	(79.93–82.26)	102.78	(101.25–104.30)	11,613,652	100.56	(98.74–102.4)	124.69	(122.33–127.05)	11,341,740	61.16	(59.72–62.60)	80.85	(78.90–82.79)
2006	23,086,319	85.74	(84.54–86.93)	106.15	(104.63–107.68)	11,665,482	104.08	(102.2–105.9)	126.76	(124.42–129.09)	11,420,837	67.00	(65.50–68.50)	85.53	(83.58–87.49)
2007	23,202,310	90.75	(89.53–91.98)	109.46	(107.94–110.97)	11,708,220	109.14	(107.2–111.0)	129.63	(127.32–131.95)	11,494,090	72.03	(70.48–73.58)	89.27	(87.31–91.22)
2008	23,309,090	96.97	(95.70–98.23)	114.03	(112.52–115.55)	11,745,723	114.54	(112.6–116.5)	132.96	(130.66–135.26)	11,563,367	79.11	(77.49–80.73)	95.10	(93.12–97.07)
2009	23,410,304	102.72	(101.4–104.0)	117.73	(116.22–119.24)	11,778,394	119.86	(117.9–121.8)	135.94	(133.66–138.22)	11,631,910	85.36	(83.68–87.04)	99.51	(97.53–101.5)
2010	23,509,764	107.08	(105.8–108.4)	119.55	(118.06–121.04)	11,810,506	121.80	(119.8–123.8)	134.56	(132.34–136.78)	11,699,258	92.22	(90.48–93.96)	104.53	(102.5–106.5)
2011	23,663,066	111.56	(110.2–112.9)	121.47	(120.00–122.95)	11,873,718	124.77	(122.8–126.8)	134.83	(132.65–137.01)	11,789,348	98.26	(96.47–100.0)	108.11	(106.1–110.1)
2012	23,751,297	118.24	(116.9–119.6)	125.23	(123.76–126.70)	11,905,497	129.39	(127.3–131.4)	136.30	(134.14–138.45)	11,845,800	107.04	(105.2–108.9)	114.15	(112.2–116.1)
2013	23,797,477	124.89	(123.5–126.3)	128.46	(127.00–129.92)	11,916,406	134.22	(132.1–136.3)	137.71	(135.58–139.85)	11,881,071	115.53	(113.6–117.5)	119.21	(117.2–121.2)
2014	23,850,842	132.40	(130.9–133.9)	132.40	(130.94–133.86)	11,930,181	139.29	(137.2–141.4)	139.29	(137.17–141.40)	11,920,661	125.50	(123.5–127.5)	125.50	(123.5–127.5)

**Table 3:** Crude and age-standardized incidence (per 100,000 person-years) of lung cancer in Taiwan from 2002 to 2014.

Year	Total					Male					Female				
	Person-years	Crude		Standardized		Person-years	Crude		Standardized		Person-years	Crude		Standardized	
2002	22,013,202	33.20	(32.44–33.96)	45.04	(43.95–46.13)	11,117,309	43.89	(42.66–45.12)	57.50	(55.77–59.22)	10,895,893	22.29	(21.41–23.18)	32.57	(31.24–33.91)
2003	22,332,502	32.91	(32.16–33.66)	43.80	(42.75–44.85)	11,303,661	43.92	(42.70–45.15)	56.84	(55.16–58.51)	11,028,841	21.63	(20.76–22.49)	30.76	(29.49–32.03)
2004	22,545,306	36.48	(35.69–37.27)	47.18	(46.12–48.25)	11,412,685	48.81	(47.52–50.09)	61.61	(59.91–63.31)	11,132,621	23.85	(22.94–24.76)	32.75	(31.48–34.03)
2005	22,693,144	37.69	(36.89–38.48)	47.70	(46.65–48.75)	11,477,894	49.77	(48.47–51.06)	61.36	(59.70–63.02)	11,215,251	25.32	(24.39–26.25)	34.03	(32.75–35.31)
2006	22,826,848	39.03	(38.22–39.84)	48.11	(47.08–49.14)	11,532,338	50.73	(49.43–52.03)	61.36	(59.72–62.99)	11,294,510	27.08	(26.12–28.04)	34.87	(33.61–36.12)
2007	22,956,217	41.22	(40.39–42.05)	49.44	(48.42–50.46)	11,583,771	53.05	(51.72–54.37)	62.52	(60.91–64.13)	11,372,446	29.18	(28.18–30.17)	36.36	(35.10–37.62)
2008	23,069,398	41.64	(40.80–42.47)	48.59	(47.60–49.58)	11,625,147	53.76	(52.43–55.10)	61.78	(60.22–63.35)	11,444,251	29.32	(28.32–30.31)	35.39	(34.17–36.60)
2009	23,174,474	43.57	(42.72–44.41)	49.73	(48.75–50.72)	11,661,944	54.74	(53.40–56.08)	61.76	(60.22–63.30)	11,512,530	32.24	(31.21–33.28)	37.70	(36.48–38.93)
2010	23,273,618	43.13	(42.28–43.97)	47.85	(46.91–48.80)	11,693,729	54.23	(52.90–55.57)	59.47	(57.99–60.95)	11,579,889	31.91	(30.88–32.94)	36.24	(35.06–37.41)
2011	23,361,690	44.99	(44.13–45.85)	48.59	(47.66–49.53)	11,719,356	56.28	(54.92–57.64)	60.33	(58.87–61.79)	11,642,334	33.62	(32.57–34.67)	36.86	(35.70–38.01)
2012	23,509,900	47.14	(46.26–48.02)	49.78	(48.85–50.71)	11,780,425	56.85	(55.49–58.21)	59.72	(58.28–61.15)	11,729,475	37.39	(36.29–38.50)	39.84	(38.66–41.02)
2013	23,595,308	47.58	(46.70–48.46)	48.86	(47.96–49.77)	11,811,225	57.67	(56.30–59.03)	59.07	(57.66–60.47)	11,784,083	37.47	(36.36–38.57)	38.66	(37.52–39.80)
2014	23,633,148	49.86	(48.96–50.76)	49.86	(48.96–50.76)	11,817,577	58.76	(57.38–60.14)	58.76	(57.38–60.14)	11,815,571	40.96	(39.81–42.12)	40.96	(39.81–42.12)

**Table 4:** Joinpoint analysis of lung cancer prevalence by sex in Taiwan from 2002 to 2014.

	Lung cancer prevalence (per 100,000 people)				Average APC	Trend 1		Trend 2	
	2002		2014			Years	APC (95% CI)	Years	APC (95% CI)
Prevalence									
Total	93.38	(91.84–94.92)	132.40	(130.94–133.86)	3.1 (2.7–3.4)^*∗*^	2002 to 2008	3.6 (3.1–4.2)^*∗*^	2008 to 2014	2.5 (2.0–2.9)^*∗*^
Male	114.95	(112.56–117.35)	139.29	(137.17–141.40)	1.7 (1.3–2.0)^*∗*^	2002 to 2008	2.7 (2.1–3.3)^*∗*^	2008 to 2014	0.6 (0.1–1.1)^*∗*^
Female	71.79	(69.85–73.74)	125.50	(123.50–127.50)	5.0 (4.8–5.1)^*∗*^	2002 to 2014	5.0 (4.8–5.1)^*∗*^		

APC, annual percent change; ^*∗*^*p* < 0.05.

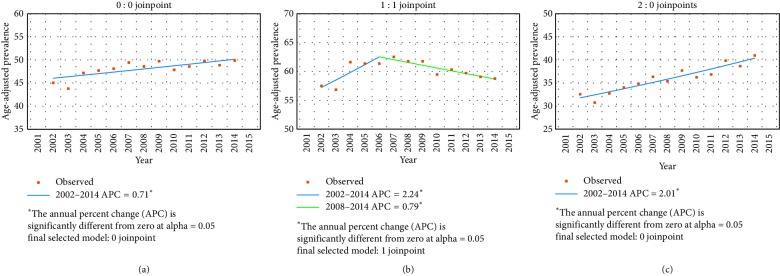

**Table 5:** Joinpoint analysis of lung cancer incidence by sex in Taiwan from 2002 to 2014.

	Lung cancer incidence (per 100,000 person-years)				Average APC	Trend 1		Trend 2	
	2002		2014			Years	APC (95% CI)	Years	APC (95% CI)
Incidence									
Total	45.04	(43.95–46.13)	49.86	(48.96–50.76)	0.7 (0.3–1.1)^*∗*^	2002 to 2014	0.7 (0.3–1.1)^*∗*^		
Male	57.50	(55.77–59.22)	58.76	(57.38–60.14)	0.2 (−0.4–0.8)	2002 to 2006	2.2 (0.5–4.0)^*∗*^	2006 to 2014	−0.8 (−1.3 to −0.3)^*∗*^
Female	32.57	(31.24–33.91)	40.96	(39.81–42.12)	2.0 (1.5–2.5)^*∗*^	2002 to 2014	2.0 (1.5–2.5)^*∗*^		

APC, annual percent change; ^*∗*^*p* < 0.05.

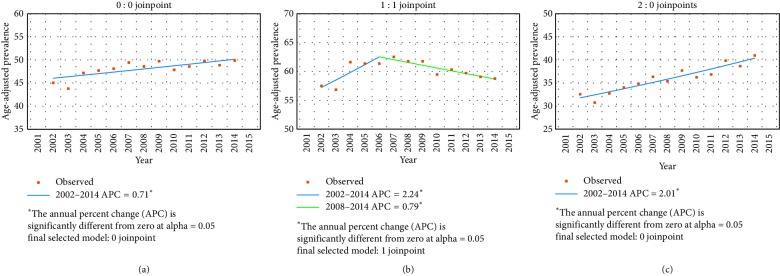

**Table 6:** Joinpoint analysis of lung cancer overall survival^a^ by sex and Charlson Comorbidity Index (CCI) in Taiwan from 2002 to 2014.

	Lung cancer survival rate				Average APC	Trend 1		Trend 2	
	2002		End^b^			Years	APC (95% CI)	Years	APC (95% CI)
Total									
1-year survival rate	37.81	(36.70–38.92)	57.39	(56.49–58.28)	3.60 (3.20–4.10)^*∗*^	2002 to 2014	3.60 (3.20–4.10)^*∗*^		
3-year survival rate	14.66	(13.86–15.48)	29.55	(28.70–30.40)	7.50 (6.50–8.60)^*∗*^	2002 to 2012	7.50 (6.50–8.60)^*∗*^		
5-year survival rate	9.37	(8.72–10.06)	17.34	(16.61–18.09)	9.30 (7.60–11.10)^*∗*^	2002 to 2010	9.30 (7.60–11.10)^*∗*^		
Sex									
Male									
1-year survival rate	34.90	(33.55–36.22)	48.20	(47.01–49.36)	3.10 (2.70–3.50)^*∗*^	2002 to 2014	3.10 (2.70–3.50)^*∗*^		
3-year survival rate	13.10	(12.13–14.02)	22.00	(20.99–22.97)	5.90 (4.60–7.30)^*∗*^	2002 to 2012	5.90 (4.60–7.30)^*∗*^		
5-year survival rate	8.40	(7.67–9.22)	12.60	(11.82–13.46)	6.30 (4.00–8.60)^*∗*^	2002 to 2010	6.30 (4.00–8.60)^*∗*^		
Female									
1-year survival rate	43.70	(41.70–45.64)	70.60	(69.29–71.86)	4.10 (3.00–5.30)^*∗*^	2002 to 2007	6.30 (3.30–9.40)^*∗*^	2007 to 2014	2.60 (1.60–3.60)^*∗*^
3-year survival rate	17.90	(16.37–19.42)	41.10	(39.66–42.57)	8.10 (7.00–9.30)^*∗*^	2002 to 2012	8.10 (7.00–9.30)^*∗*^		
5-year survival rate	11.30	(10.06–12.58)	25.40	(24.04–26.85)	11.80 (10.50–13.00)^*∗*^	2002 to 2010	11.80 (10.50–13.00)^*∗*^		

CCI									
CCI ≤ 1									
1-year survival rate	42.40	(39.98–44.82)	69.80	(68.33–71.20)	4.00 (3.50–4.50)^*∗*^	2002 to 2014	4.00 (3.50–4.50)^*∗*^		
3-year survival rate	17.70	(15.85–19.59)	39.20	(37.58–40.79)	8.60 (6.90–10.20)^*∗*^	2002 to 2012	8.60 (6.90–10.20)^*∗*^		
5-year survival rate	11.40	(9.93–13.05)	24.50	(22.98–26.04)	11.30 (9.00–13.80)^*∗*^	2002 to 2010	11.30 (9.00–13.80)^*∗*^		
CCI ≥ 2									
1-year survival rate	36.50	(35.27–37.77)	51.20	(50.08–52.29)	3.10 (2.60–3.60)^*∗*^	2002 to 2014	3.10 (2.60–3.60)^*∗*^		
3-year survival rate	13.80	(12.93–14.72)	25.00	(24.05–26.00)	6.40 (5.60–7.20)^*∗*^	2002 to 2012	6.40 (5.60–7.20)^*∗*^		
5-year survival rate	8.80	(8.08–9.55)	14.30	(13.44–15.08)	7.40 (5.80–9.00)^*∗*^	2002 to 2010	7.40 (5.80–9.00)^*∗*^		

APC, annual percent change; CCI, Charlson Comorbidity Index; ^*∗*^*p* < 0.05. ^a^Overall survival of patients with lung cancer was extracted from overall mortality rates in the National Death Registry of Taiwan, and the relative survival rates were estimated using the life table method. ^b^The end year in one-year survival is 2014, the end year in three-year survival is 2012, and the end year in five-year survival is 2010.

**Table 7:** Clinical characteristics of lung cancer patients with different histological subtypes in Taiwan from 2002 to 2014.

	Large cell carcinoma (*N* = 1,319)		Small cell carcinoma (*N* = 10,582)		Adenocarcinoma (*N* = 67,649)		Squamous cell carcinoma (*N* = 22,951)		Other types of lung cancer (*N* = 21,647)	
Age (years) (mean ± standard deviation)	65.66 ± 12.91		69.90 ± 11.00		66.43 ± 12.96		70.89 ± 11.13		72.07 ± 13.17	
Sex										
Female	393	29.8	1,150	10.87	33,584	49.64	2,940	12.81	6,936	32.04
Male	926	70.2	9,432	89.13	34,065	50.36	20,011	87.19	14,711	67.96
Place of residence, no. (%)										
Urban	660	(50.04)	4,953	(46.81)	36,765	(54.35)	10,020	(43.66)	9,789	(45.22)
Suburban	394	(29.87)	3,170	(29.96)	19,560	(28.91)	7,424	(32.35)	6,325	(29.22)
Rural	147	(11.14)	1,356	(12.81)	7,284	(10.77)	3,456	(15.06)	2,657	(12.27)
Unknown	118	(8.95)	1,103	(10.42)	4,040	(5.97)	2,051	(8.94)	2,876	(13.29)
Income levels, no. (%)										
Quintile 1	176	(13.34)	1,668	(15.76)	7,817	(11.56)	3,388	(14.76)	3,487	(16.11)
Quintile 2	311	(23.58)	2,673	(25.26)	16,752	(24.76)	6,196	(27.0)	5,700	(26.33)
Quintile 3	157	(11.9)	1,458	(13.78)	8,354	(12.35)	3,406	(14.84)	2,815	(13.0)
Quintile 4	309	(23.43)	2,127	(20.1)	15,674	(23.17)	4,861	(21.18)	3,563	(16.46)
Quintile 5	253	(19.18)	1,568	(14.82)	15,277	(22.58)	3,120	(13.59)	3,251	(15.02)
Unknown	113	(8.57)	1,088	(10.28)	3,775	(5.58)	1,980	(8.63)	2,831	(13.08)
Occupation, no. (%)										
Dependents of the insured individuals	369	(27.98)	2,938	(27.76)	21,225	(31.38)	6,210	(27.06)	6,054	(27.97)
Civil servants, teachers, military personnel, and veterans	50	(3.79)	446	(4.21)	3,379	(4.99)	1,050	(4.57)	951	(4.39)
Nonmanual workers and professionals	100	(7.58)	450	(4.25)	6,468	(9.56)	939	(4.09)	1,033	(4.77)
Manual workers	424	(32.15)	3,335	(31.52)	20,448	(30.23)	8,184	(35.66)	6,422	(29.67)
Other	376	(28.51)	3,413	(32.25)	16,129	(23.84)	6,568	(28.62)	7,187	(33.2)
Charlson Index (mean ± standard deviation)	3.58 ± 3.01		4.02 ± 3.14		3.68 ± 3.09		3.58 ± 2.86		4.29 ± 3.22	

**Table 8:** Joinpoint analysis of overall survival^a^ for lung cancer patients with different histological subtypes in Taiwan during 2002–2014.

	Lung cancer survival rate				Average APC	Trend 1		Trend 2	
	2002		End^b^			Years	APC (95% CI)	Years	APC (95% CI)
Large cell carcinoma									
1-year survival rate	40.60	(28.61–52.29)	58.10	(48.88–66.18)	2.60 (1.00–4.10)^*∗*^	2002 to 2014	2.60 (1.00–4.10)^*∗*^		
3-year survival rate	21.90	(12.73–32.61)	36.80	(28.42–45.18)	4.70 (2.30–7.20)^*∗*^	2002 to 2012	4.70 (2.30–7.20)^*∗*^		
5-year survival rate	20.30	(11.52–30.86)	30.20	(21.76–39.04)	5.80 (0.20–11.70)^*∗*^	2002 to 2010	5.80 (0.20–11.70)^*∗*^		
Small cell carcinoma									
1-year survival rate	27.40	(23.98–30.83)	29.10	(26.08–32.16)	1.70 (0.60–2.70)^*∗*^	2002 to 2014	1.70 (0.60–2.70)^*∗*^		
3-year survival rate	5.60	(3.98–7.52)	5.30	(3.90–6.94)	2.20 (−0.30–4.70)	2002 to 2012	2.20 (−0.30–4.70)		
5-year survival rate	3.10	(1.95–4.64)	4.40	(3.12–5.91)	5.40 (2.10–8.80)^*∗*^	2002 to 2010	5.40 (2.10–8.80)^*∗*^		
Adenocarcinoma									
1-year survival rate	44.70	(42.96–46.42)	67.90	(66.81–68.90)	3.70 (2.80–4.70)^*∗*^	2002 to 2007	5.50 (3.10–7.90)^*∗*^	2007 to 2014	2.50 (1.70–3.30)^*∗*^
3-year survival rate	17.70	(16.41–19.07)	38.30	(37.15–39.45)	7.60 (6.20–8.90)^*∗*^	2002 to 2012	7.60 (6.20–8.90)^*∗*^		
5-year survival rate	11.10	(10.08–12.27)	22.00	(20.93–23.07)	10.60 (8.40–12.90)^*∗*^	2002 to 2010	10.60 (8.40–12.90)^*∗*^		
Squamous cell carcinoma									
1-year survival rate	38.50	(36.11–40.85)	43.00	(40.72–45.24)	1.20 (0.70–1.60)^*∗*^	2002 to 2014	1.20 (0.70–1.60)^*∗*^		
3-year survival rate	15.60	(13.85–17.38)	17.40	(15.65–19.14)	2.10 (0.80–3.50)^*∗*^	2002 to 2012	2.10 (0.80–3.50)^*∗*^		
5-year survival rate	10.60	(9.18–12.18)	12.90	(11.37–14.46)	3.10 (−0.40–6.80)	2002 to 2008	0.30 (−2.80–3.60)	2008 to 2010	12.00 (−5.60–33.00)
Other subtypes of lung cancer									
1-year survival rate	28.70	(26.63–30.81)	35.00	(32.43–37.58)	1.40 (0.70–2.10)^*∗*^	2002 to 2014	1.40 (0.70–2.10)^*∗*^		
3-year survival rate	11.40	(10.02–12.96)	16.40	(14.53–18.36)	4.10 (2.50–5.80)^*∗*^	2002 to 2012	4.10 (2.50–5.80)^*∗*^		
5-year survival rate	7.00	(5.88–8.24)	11.60	(10.07–13.21)	5.00 (2.00–8.20)^*∗*^	2002 to 2010	5.00 (2.00–8.20)^*∗*^		

APC, annual percent change; ^*∗*^*p* < 0.05. ^a^Overall survival of patients with lung cancer were extracted from overall mortality rates in the National Death Registry of Taiwan, and the relative survival rates were estimated using the life table method. ^b^The end year in one-year survival is 2014, the end year in three-year survival is 2012, and the end year in five-year survival is 2010.

## Data Availability

The data used to support the findings of this study are available from the corresponding author upon request.
